# Supplementary material for: Tunable giant magnetoresistance in a single-molecule junction
Source: Nat Commun. 2019 Aug 9;10:3599. doi: 10.1038/s41467-019-11587-x (PMC6689026; doi:10.1038/s41467-019-11587-x)
Supplement: Supplementary file 1 — Supplementary Information [file 41467_2019_11587_MOESM1_ESM.pdf]

Supplementary Information

Tunable giant magnetoresistance in a single-molecule junction

Yang *et al*

Supplementary Information

Tunable giant magnetoresistance in a single-molecule junction

Kai Yang[1†], Hui Chen[1†], Thomas Pope[2†], Yibin Hu[3†], Liwei Liu[1], Dongfei Wang[1], Lei Tao[1], Wende Xiao[1], Xiangmin Fei[1], Yu-Yang Zhang[1], Hong-Gang Luo[4], Shixuan Du[1], Tao Xiang[1], Werner A. Hofer[1,2]* and Hong-Jun Gao[1]*

[1]Institute of Physics & University of Chinese Academy of Sciences, Chinese Academy of Sciences, Beijing 100190, China

[2]School of Natural and Environmental Sciences, Newcastle University, Newcastle NE1 7RU, UK

[3]State Key Laboratory of Infrared Physics, Shanghai Institute of Technical Physics, Chinese Academy of Sciences, Shanghai 200083, China

[4]School of Physical Science and Technology, Lanzhou University, Lanzhou 730000, China

*Corresponding authors: H.-J. G. (hjgao@iphy.ac.cn) and W.A.H. (Werner.Hofer@newcastle.ac.uk)

Table of contents

Supplementary Note 1. Configurations of FePc on Au(111) ... 3

Supplementary Note 2. Temperature dependence of the dI/dV spectra taken on FePc (I) ... 5

Supplementary Note 3. Spatial distribution of the Kondo resonance at different magnetic field ... 6

Supplementary Note 4. Fitting dI/dV spectra under magnetic field ... 7

Supplementary Note 5. dI/dV spectra of FePc (I) on Au(111) showing Zeeman splittings ... 8

Supplementary Note 6. dI/dV spectra and mapping of an Fe-porphyrin derivative on Au(111) ... 9

Supplementary Note 7. Magnetization vector on the Fe atom ... 9

Supplementary Note 8. Broad dI/dV feature ... 10

Supplementary References ... 11

Supplementary Note 1. Configurations of FePc on Au(111)

After adsorption on Au(111), each FePc molecule appears as a cross with a central protrusion in STM topographies (Supplementary Fig. 1a). We identified two non-equivalent molecular adsorption configurations (labeled I and II) on Au(111), as revealed by detailed simulations[1]. The Fe ion is located at a bridge (on-top) site with respect to Au(111) substrate in configuration I (II). In the bridge configuration, the cross structure of FePc is oriented parallel to the <1$\bar{1}$0> and <11$\bar{2}$> directions of Au(111), while it rotates by approximately 15° in the on-top configuration. The apparent height of the central protrusion of FePc (II) is lower than that of FePc (I) (Supplementary Fig. 1a).

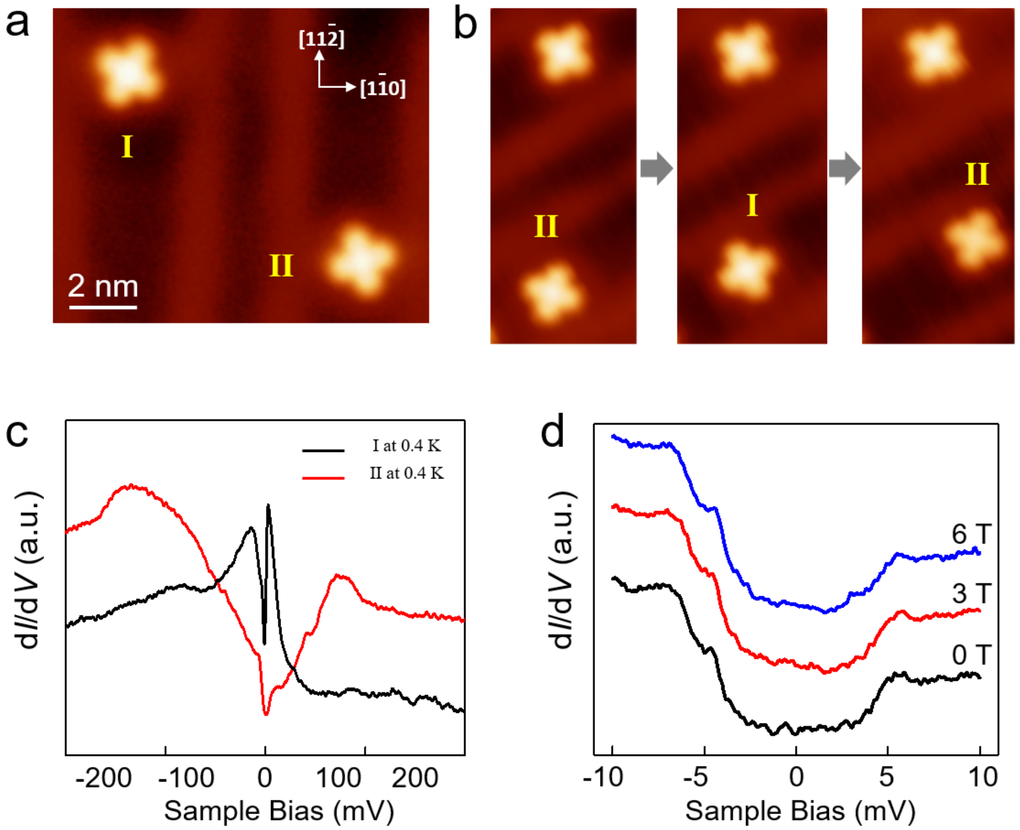

Supplementary Fig. 1. **a,** STM image (I = 10 pA, V_b= −0.2 V) of isolated FePc molecules in the fcc region of Au(111), showing two types of adsorption configurations. **b,** Switching the configuration of a single FePc molecule in the fcc region of Au(111). By sequential lateral manipulation with STM tip, FePc (II) is switched to FePc (I) and then back to FePc (II). Image size: 3 nm × 7 nm. **c,** dI/dV spectra (setpoint: I = 0.2 nA, V_b= −0.1 V) taken on the Fe ions for both configurations at 0.4 K, showing dramatically different characteristic features near E_F: a dip superimposed on a broad feature for FePc (I) and broad dip with fine features for FePc (II). **d,** dI/dV spectra (setpoint: I = 0.3 nA, V_b= −80 mV) of FePc (II) in the fcc region measured on Fe ion under different magnetic field at 0.4 K. Successive spectra are offset for clarity.

We note that the decoration of foreign atoms or molecules (such as H and CO) on metal phthalocyanine molecules can modify their topographic and electronic structures[2,3]. To exclude this possibility in our experiment, we dragged a single FePc molecule along the fcc region of Au(111) surface and found that its configuration could be switched between I and II (Supplementary Fig. 1b), showing characteristic spectral features (Supplementary Fig. 1c), respectively. Hence, the difference in spectral features between I and II is not caused by foreign atoms or molecules decorated on FePc.

Differential conductance (dI/dV) spectra taken on Fe ions of the two configurations in the fcc regions of Au(111)[4] exhibit dramatically different features near Fermi level (E_F), as shown in Supplementary Fig. 1c. The configuration discussed in the main text is FePc (I). For FePc (II), no dip-to-peak transition is observed under magnetic fields. Instead, inelastic electron tunneling spectra (IETS) are observed (Supplementary Fig. 1d). The independence of the step positions with magnetic field suggests that the IETS features may come from vibrational excitations[5].

Our previous studies of FePc at the bridge site did not exhibit the sharp Kondo resonance in the dI/dV spectra[1]. This is due to the limited energy resolution, resulting from the broadening by the relatively large modulation voltage and noise originating from the electrical setup, together acting as an effective high noise temperature.

Supplementary Note 2. Temperature dependence of the dI/dV spectra taken on FePc (I)

From the temperature-dependent dI/dV spectra (Fig. 2b, main text), we determined the Kondo temperature to be T_K = 5.2 ± 0.2 K (Supplementary Fig. 2b). We also measured the spectra at 4.2 K under different magnetic fields (Supplementary Fig. 2c), and observed a similar dip-to-peak transition as in the spectra taken at 0.4 K.

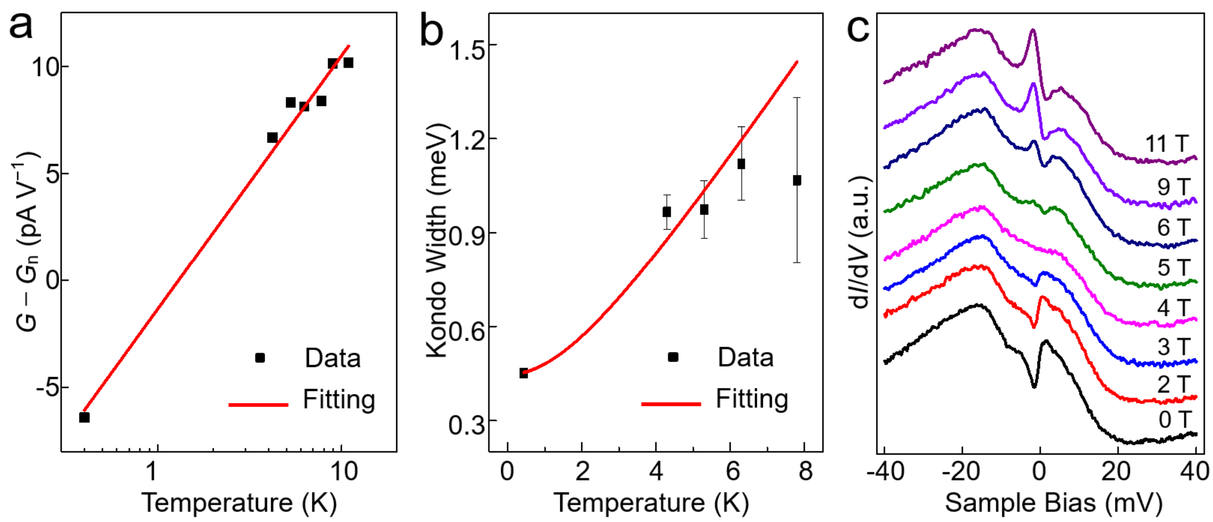

Supplementary Fig. 2 **a,** Temperature dependence of the difference between the zero-bias conductance (G) and the conductance measured at 40 meV (G_n), $G-G_n$ (corresponding to Fig. 2b in the main text). The solid curve is a logarithmic fit to the data. **b,** Temperature dependence of the half-width at half-maximum (HWHM) of the Kondo resonance. The HWHM is extracted by fitting the normalized dI/dV data (by subtracting the dI/dV spectrum at 8.9 K) using the Fano equation. The solid line is a fitting curve using $\mathrm{HWHM} = \frac{1}{2}\sqrt{(\alpha k_B T)^2 + (2k_B T_K)^2}$ according to Ref. [6], which gives α = 4.1 ± 0.3 and T_K = 5.2 ± 0.2 K. Error bars are the fitting uncertainties of the Kondo line width with a 95% confidence. **c,** dI/dV spectra (setpoint: I = 0.3 nA, V_b = −60 mV) for FePc (I) in the fcc region measured on Fe ion under different magnetic fields at 4.2 K. Successive spectra are offset for clarity.

Supplementary Note 3. Spatial distribution of the Kondo resonance at different magnetic field

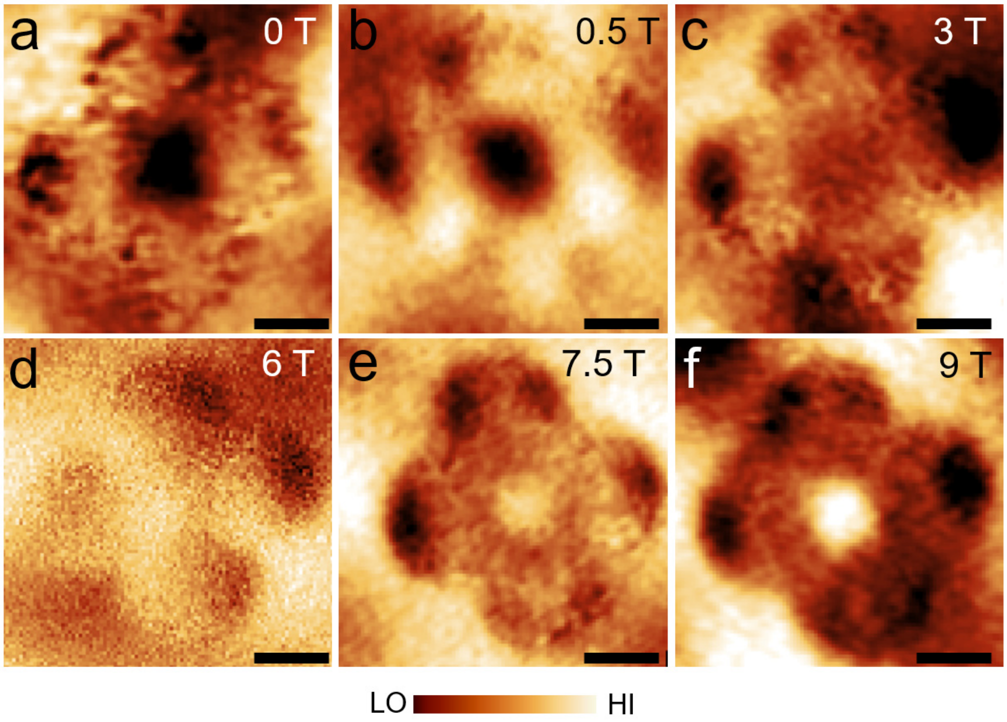

Supplementary Fig. 3 **a,** dI/dV mapping taken around the Fermi energy at zero magnetic field (setpoint: I = 0.3 nA, V_b = −40 mV). **b-f,** dI/dV mapping at 0.5 T, 3 T, 6 T, 7.5 T and 9 T, respectively. At 9 T the shape has changed to a more localized circular protrusion as shown by the green dashed curve in **f**. Scale bar: 0.5 nm.

Supplementary Note 4. Fitting dI/dV spectra under magnetic field

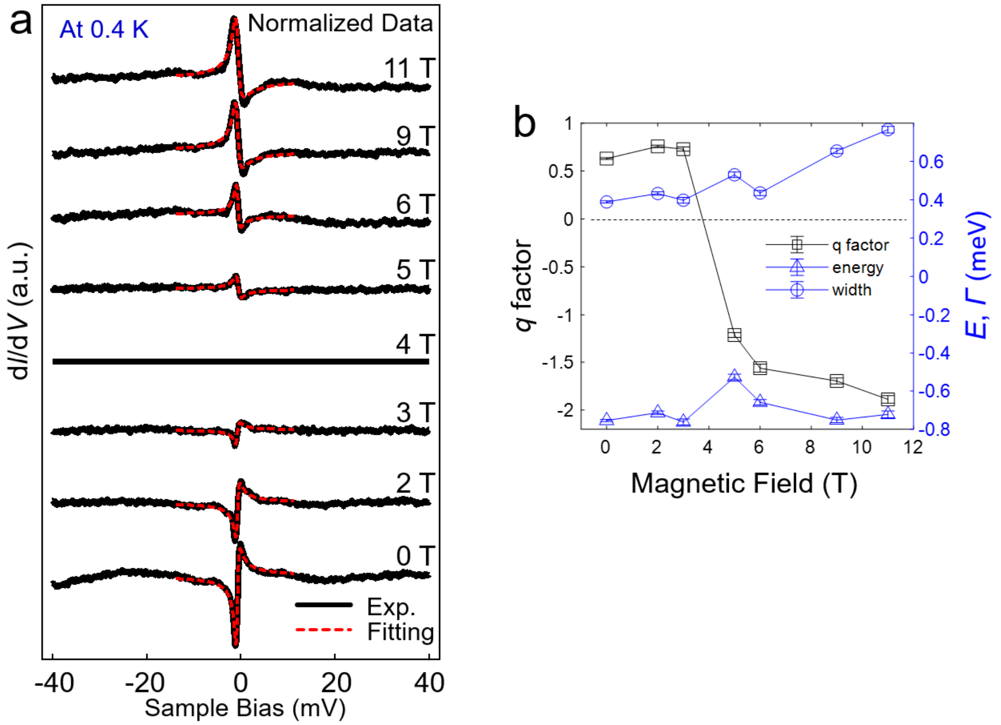

Supplementary Fig. 4 **a,** Normalized dI/dV spectra by subtracting the dI/dV spectrum at 4 T to remove the broad background. Red dashed lines show the Fano fitting: $\frac{dI}{dV}(V) = A \cdot \frac{(\varepsilon+q)^2}{1+\varepsilon^2} + B, \varepsilon = (eV - \varepsilon_0)/\Gamma$, where q is Fano asymmetry factor, ε_0 is the position of the resonance and Γ is the half-width at half-maximum of the Kondo resonance. **b,** Magnetic field dependence of the Fano fitting parameters: energy position, width and q factor of the normalized Kondo resonance. Error bars are the fitting uncertainties of the Fano line shapes in **a**.

Supplementary Note 5. dI/dV spectra of FePc (I) on Au(111) showing Zeeman splittings

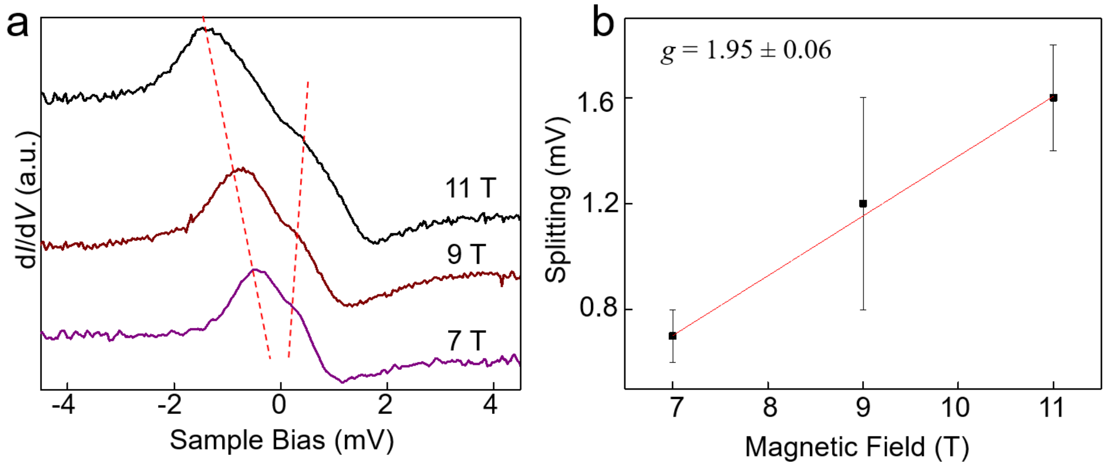

Supplementary Fig. 5 **a,** dI/dV spectra of FePc (I) under different magnetic fields at 0.4 K (setpoint: I = 0.7 nA, V_b = −10 mV), clearly showing the Zeeman splitting near E_F for B ≥ 7 T. Successive spectra are offset for clarity. **b,** Zeeman splitting as a function of magnetic field. A linear fit (red line) yields g = 1.95 ± 0.06. Error bars are propagated from the fitting uncertainties of the Kondo peak positions in **a**.

Supplementary Note 6. d*I*/d*V* spectra and mapping of an Fe-porphyrin derivative on Au(111)

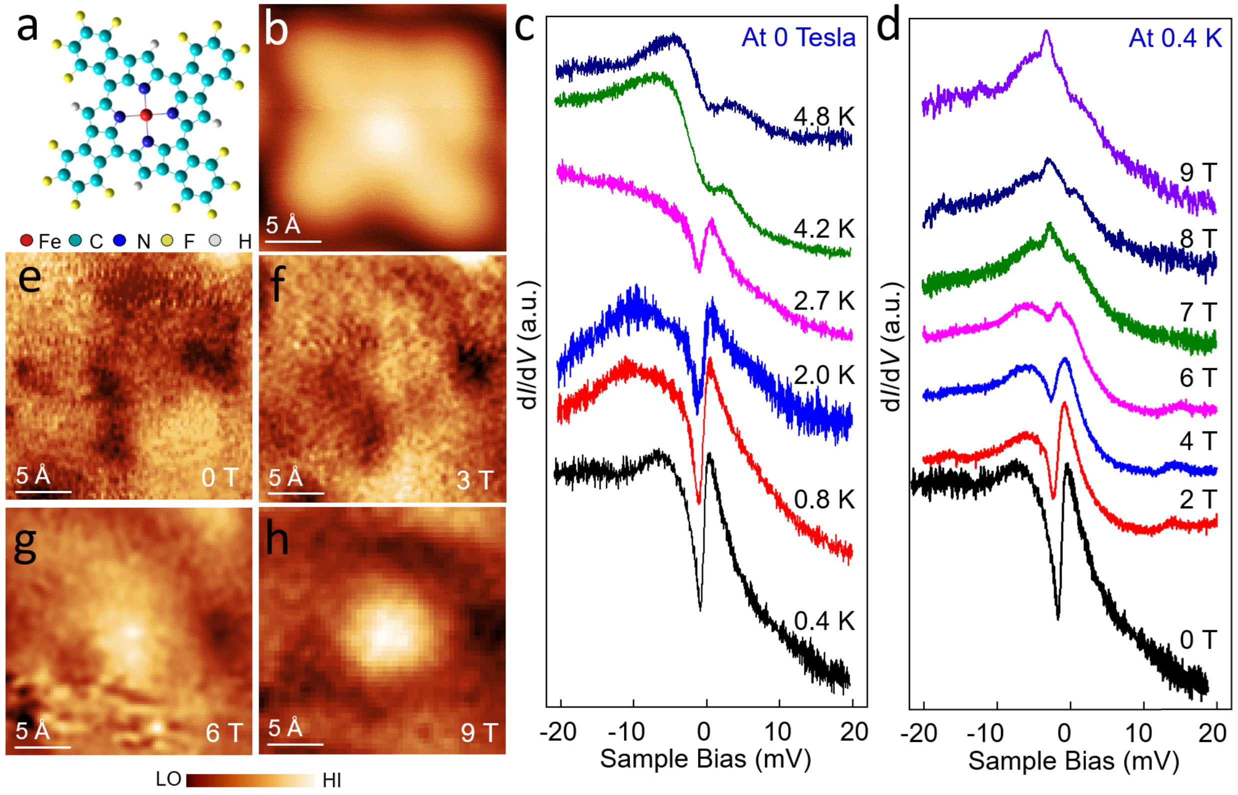

Supplementary Fig. 6 d*I*/d*V* spectra and mapping of an Fe-porphyrin derivative on Au(111). **a,** molecular model of the Fe-porphyrin derivative. **b,** STM image (2.2 nm × 2.2 nm, I = 10 pA, V_b = −0.2 V) of the Fe-porphyrin derivative. **c,** Evolution of the d*I*/d*V* spectra (setpoint: I = 0.3 nA, V_b = −40 mV) of the Fe-porphyrin derivative at various temperatures in the absence of magnetic field. Successive spectra are offset for clarity. **d,** Evolution of the d*I*/d*V* spectra (setpoint: I = 0.3 nA, V_b = −40 mV) of the Fe-porphyrin derivative with increasing magnetic field, showing a dip-to-peak transition. **e-h,** d*I*/d*V* mapping taken around the Fermi energy at 0 T, 3 T, 6 T and 9 T (2.2 nm × 2.2 nm, setpoint: I = 0.3 nA, V_b = −40 mV) showing a transition from an extended distribution to a more concentric distribution.

Supplementary Note 7. Magnetization vector on the Fe atom

We consider two electronic configurations; the first with near in-plane anisotropy and the second with a magnetization vector on the Fe atom aligned perpendicular to the plane of the molecule. The ground state of the FePc molecule is an unresolved problem. Magnetic anisotropy experiments originally assigned the ^3E_g multiplet as the ground state[7,8], but later magnetic susceptibility and magnetic circular dichroism experiments favored either the $^3A_{2g}$ or $^3B_{2g}$ configurations[9,10]. In addition, it has been proposed that the ground state might be a mix of the $^3E_{2g}$

and $^3B_{2g}$ configurations[11-13]. Typically, DFT studies predict the $^3A_{2g}$ configuration, but recent studies have shown that the $^3B_{2g}$ and ^3E_g are also predicted depending on the choice of exchange-correlation functional[14,15]. In our case, the $^3A_{2g}$ configuration is chosen for the ground state, corresponding to a partially filled d_π orbital, and thus leading to the in-plane anisotropy. In order to find this ground state magnetization vector **m**, we sample a range of initial configurations, both in plane and out of plane, and performed an unconstrained non-collinear calculation. We define **i** and **j** as the unit vectors in the molecular plane and **k** perpendicular to the molecular plane. After an initial guess of $\mathbf{m} = \mathbf{i}+\mathbf{j}$, the lowest energy configuration we found was $\mathbf{m} = 1.217\mathbf{i}+1.184\mathbf{j}+1.157\mathbf{k}$, which we took to be the ground state configuration at low magnetic field. We also initialized the system with a magnetization vector $\mathbf{m} = -\mathbf{k}$, which minimized to $m = -0.027\mathbf{i}-0.017\mathbf{j}-3.708\mathbf{k}$. We took this to be the aligned configuration at high magnetic field.

Supplementary Note 8. Broad d*I*/d*V* feature

To explain the broader feature seen in the d*I*/d*V* spectra, we calculate the density of states for the system with the in-plane magnetization. We find that the DOS exhibits the same two-peak structure seen in the broad d*I*/d*V* feature (Supplementary Fig. 7).

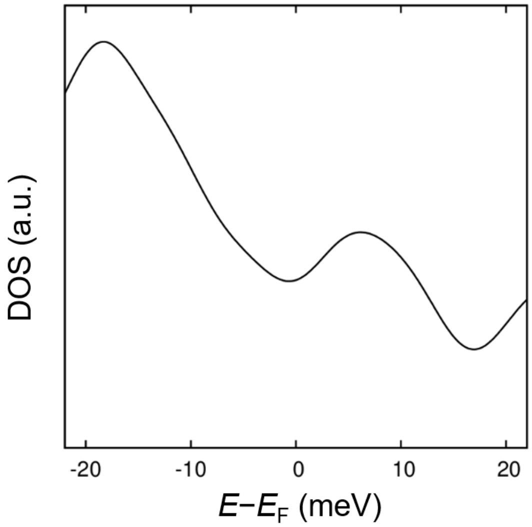

Supplementary Fig. 7 Calculated DOS of FePc on the gold surface with an in-plane magnetization vector.

Supplementary References

1 L. Gao *et al.* Site-specific Kondo effect at ambient temperatures in iron-based molecules. *Phys. Rev. Lett.* **99**, 106402 (2007).

2 A. Stróżecka, M. Soriano, J. I. Pascual & J. J. Palacios. Reversible change of the spin state in a manganese phthalocyanine by coordination of CO molecule. *Phys. Rev. Lett.* **109**, 147202 (2012).

3 L. W. Liu *et al.* Reversible single spin control of individual magnetic molecule by hydrogen atom adsorption. *Sci. Rep.* **3**, 1210 (2013).

4 J. V. Barth, H. Brune, G. Ertl & R. J. Behm. Scanning tunneling microscopy observations on the reconstructed Au(111) surface: Atomic structure, long-range superstructure, rotational domains, and surface defects. *Phys. Rev. B* **42**, 9307-9318 (1990).

5 B. C. Stipe, M. A. Rezaei & W. Ho. Single-molecule vibrational spectroscopy and microscopy. *Science* **280**, 1732-1735 (1998).

6 K. Nagaoka, T. Jamneala, M. Grobis & M. F. Crommie. Temperature dependence of a single Kondo impurity. *Phys. Rev. Lett.* **88**, 077205 (2002).

7 B. W. Dale, R. J. P. Williams, C. E. Johnson & T. L. Thorp. S = 1 spin state of divalent iron. I. Magnetic properties of phthalocyanine iron (ii). *J. Phys. Chem.* **49**, 3441-3444 (1968).

8 P. Coppens, L. Li & N. J. Zhu. Electronic ground state of iron(ii) phthalocyanine as determined from accurate diffraction data. *J. Am. Chem. Soc.* **105**, 6173-6174 (1983).

9 C. G. Barraclough, R. L. Martin, S. Mitra & R. C. Sherwood. Paramagnetic anisotropy, low temperature magnetization, and electronic structure of iron(ii) phthalocyanine. *J. Phys. Chem.* **53**, 1643-1648 (1970).

10 M. J. Stillman & A. J. Thomson. Assignment of the charge-transfer bands in some metal phthalocyanines. Evidence for the s = 1 state of iron (ii) phthalocyanine in solution. *J. Chem. Soc., Faraday Trans. 2* **70**, 790-804 (1974).

11 S. Stepanow *et al.* Mixed-valence behavior and strong correlation effects of metal phthalocyanines adsorbed on metals. *Phys. Rev. B* **83**, 220401 (2011).

12 P. A. Reynolds & B. N. Figgis. Metal phthalocyanine ground states: Covalence and ab initio calculation of spin and charge densities. *Inorg. Chem.* **30**, 2294-2300 (1991).

13 J. Fernández-Rodríguez, B. Toby & M. van Veenendaal. Mixed configuration ground state in iron(ii) phthalocyanine. *Phys. Rev. B* **91**, 214427 (2015).

14 N. Marom & L. Kronik. Density functional theory of transition metal phthalocyanines, ii: electronic structure of mnpc and FePc—symmetry and symmetry breaking. *Appl. Phys. A* **95**, 165-172 (2009).

15 T. Ichibha, Z. Hou, K. Hongo & R. Maezono. New insight into the ground state of FePc: A diffusion monte carlo study. *Sci. Rep.* **7**, 2011 (2017).
