# Supplementary material for: Tunable giant magnetoresistance in a single-molecule junction
Source: Nat Commun. 2019 Aug 9;10:3599. doi: 10.1038/s41467-019-11587-x (PMC6689026; doi:10.1038/s41467-019-11587-x)
Supplement: Supplementary file 2 — Peer Review [file 41467_2019_11587_MOESM2_ESM.pdf]

Reviewers' comments:

Reviewer #1 (Remarks to the Author):

Yang et al. present a magnetoresistance effect for the iron phthalocyanine molecule deposited on Au(111) and measured with non-magnetic STM tips. Measurements were performed at low temperature under an external magnetic field.
Magnetoresistance is best observed at lower temperature and is associated with the field dependence of the Kondo resonance, which shows two distinct regimes. The authors associate these regimes to different conducting orbitals, which is the key point of the article.

Magnetoresistance in setups without featuring any magnetic electrode are already known (Nano Lett. 2016, 16, 4960−4967 or ACS Nano 2016, 10, 8571−8577). The novelty of the article rests in the orbital dependent mechanism. Authors provide the change in spatial distribution of the Kondo resonance as the proof of such mechanism and support this interpretation by DFT calculations. From my point of view, these arguments are largely insufficient to justify the main claim of the article, so I suggest rejecting it.

Zeeman effect is associated with tiny energy splitting in comparison with orbital energy differences. Their ground state is not degenerate, so orbital energy differences in the range of meV cannot be present. This contradicts the necessity of having the d_z2 and d_pi orbitals in close proximity to allow crossover.

Even if the state were degenerate, a single reference method (as non-collinear DFT) will not map to the relevant state manifold for this case. In this way, the calculations presented by the authors are not conclusive support for the orbital switching mechanism.

Reviewer #2 (Remarks to the Author):

The authors present a very interesting experimental and theoretical work on the Kondo effect in FePc molecules adsorbed on Au(111). Depending on the adsorption site, a clear Kondo effect visible as a dip at zero bis was found. Varying the tempertaure, a Kondo effect is confiremd. Interestingly, the Kondo resonance reacts to a magnetic field in a peculiar way. At about 4 T, it vanishes and at higher fields reappears as a peak that splits in accord to a Kondo effect.

The paper is well written und clearly organized. Where necessary, additional information is given in the supplementary. Overall, the paper is scientifically sound and interesting and should be published in Nature Comm.

Before acceptance, I suggest to

1. go through the paper and improve the English a bit

2. weaken the claim about the shape of the orbitals. While at high fields, I agree that the Kondo cloud looks basically round, the shape at low field is not exactly elliptical (see for example Fig. S3a). I would also suggest to remove the oval and circle in the figures, as they rather suggest a shape and distract the reader from making his/her owen judgement on the shape. The shape of the orbital does change but I would not go so far as to identify the shape in the experiment. The theory shows the orbitals and they kind of agree with the observation. To my taste, this is enought.

List of Changes (marked by the red color in the revised text)

Main text:

1. We have added several sentences in the revised manuscript to further clarify the effect of the magnetic field on the orbital characteristics of the ground state of the FePc molecule on Au(111):

"*The change of orbital characteristics results from the reorientation of the Fe magnetic moment induced by the applied magnetic field. This reorientation changes the orbital character through spin-orbit interaction*[29]*. The applied magnetic field flips the magnetic moment of Fe from the in-plane to the out-of-plane direction when the Zeeman energy is larger than the magnetic anisotropy energy.*"

"*Similar energy scale of this magnetic anisotropy energy was obtained by other simulations*[30] *as well as by the X-ray magnetic circular dichroism measurements*[31]."

2. We have followed the reviewer's suggestions and slightly weakened the claim about the shape of the orbitals in the revised manuscript. The 'elliptical' is changed to 'non-circular' in the following sentences:

"*At zero or low fields, the Kondo resonance shows a* **non-circular** *spatial distribution (Fig. 3a).*"

"*The two different spatial distributions (***non-circular** *or circular) suggest two distinct Kondo screening channels originating from two d orbitals at different magnetic fields*."

"*…we find that the* **non-circular** *depression at low field is due to the* d_π *orbital (Fig. 4c)*."

3. Fig. 3: The circle and oval are removed in panels (a) and (d), and the corresponding description in the figure caption is removed. Figs. 3b and 3c were taken at 0 T but mislabeled as '0.5 T', and are labeled as '0 T' in the revised version. Correspondingly, the old Fig. 3a (taken at 0.5 T) is replaced by Fig. S3a (taken at 0 T).

4. Four news references are added as follows:

"*Similar energy scale of this magnetic anisotropy energy was obtained by other simulations*[30] *as well as by the X-ray magnetic circular dichroism measurements*[31]."

[30] J. Hu & R. Wu. Control of the magnetism and magnetic anisotropy of a single-molecule magnet with an electric field. Phys. Rev. Lett. 110, 097202 (2013).

[31] J. Bartolomé et al. Highly unquenched orbital moment in textured Fe-phthalocyanine thin films. Phys. Rev. B 81, 195405 (2010).

"*In addition, the TAMR effect with non-magnetic electrodes has been shown in mechanical break junctions*[41,42]."

[41] R. Hayakawa *et al*. Large magnetoresistance in single-radical molecular junctions. Nano Lett. 16, 4960 (2016).

[42] Z. Xie *et al*. Large magnetoresistance at room temperature in organic molecular tunnel junctions with nonmagnetic electrodes. ACS Nano 10, 8571 (2016).

5. The grammar and language of the manuscript have been carefully revised as below.

(1) Abstract: in the 3rd sentence, "*flow of the current*" is changed to "*flow of current*"; in the 4th sentence, "*, and*" is changed to "*or the*"; in the 8th sentence, "*itself*" is deleted.

(2) 1st paragraph: the comma is deleted in the 3rd sentence.

(3) 2nd paragraph: in the 1st sentence, "*a*" is changed to "*of*"; in the last sentence, one comma is deleted.

(4) 3rd paragraph: the first two sentences are combined by adding "*and*" and "*that*".

(5) 4th paragraph: "*it*" is added in the last sentence.

(6) 7th paragraph: in the 3rd sentence, "*with the major axis along two azamethine nitrogen atoms*" are deleted; in the 4th sentence, "*elliptical*" is deleted; in the 5th sentence, "*along the major axis of the ellipse (Fig. 3c) than the other direction*" is changed to "*along one axis of the feature (Fig. 3c) than the other*"; in the 6th sentence, "*more*" is added.

(7) 8th paragraph: in the 2nd sentence, "*field*" is changed to "*fields*"; the 3rd sentence is removed; in the last sentence, "*characters*" is changed to "*character*".

(8) 9th paragraph: "*(PDOS)*" is added in the 5th sentence.

(9) 11th paragraph: "*in*" is changed to "*is*" in the 4th sentence.

(10) 12th paragraph: the last sentence is changed from "*Unlike previous experiments, the magnetic FePc molecule is attached directly on a gold metal electrode in our tunnel junction and there is no any additional magnetic layer*." to "*Here, the magnetic FePc molecule is attached directly to a gold metal electrode in our tunnel junction and there is no additional magnetic layer*."

(11) 13th paragraph: "*Increasing magnetic field corresponds to the changes*" is changed to "*Increasing the magnetic field corresponds to a change*" in the 2nd sentence; "*voltages*" is changed to "*voltage*" in the 3rd sentence; "*TMAR*" is changed to "*TAMR*" in the last two sentences.

(12) 14th paragraph: "*there*" is added in the 2nd sentence; "*behaviour*" is changed to "*behavior*" in the 3rd sentence; "*concentric*" is changed to "*concentrated*" in the last sentence.

(13) 15th paragraph: "*a*" is deleted in the 1st sentence.

6. A data availability statement is added.

7. We updated the one grant number in Acknowledgements.

Supplementary Information:

1. Fig. S3: The circle and oval are removed, and the corresponding description in the figure caption is removed.

2. Caption of Fig. S2b. Description of the error bars is added:

"Error bars are the fitting uncertainties of the Kondo line width with a 95% confidence."

3. Caption of Fig. S4b. Description of the error bars is added:

"Error bars are propagated from the fitting uncertainties of the Kondo peak positions in a."

4. Fig. S5b: error bars are added, and one sentence is added in the caption:

"Error bars are the fitting uncertainties of the Fano line shapes in a."

Response to Reviewer #1

(1) *Yang et al. present a magnetoresistance effect for the iron phthalocyanine molecule deposited on Au(111) and measured with non-magnetic STM tips. Measurements were performed at low temperature under an external magnetic field. Magnetoresistance is best observed at lower temperature and is associated with the field dependence of the Kondo resonance, which shows two distinct regimes. The authors associate these regimes to different conducting orbitals, which is the key point of the article.*

Magnetoresistance in setups without featuring any magnetic electrode are already known (Nano Lett. 2016, 16, 4960−4967 or ACS Nano 2016, 10, 8571−8577).

Reply: We thank the reviewer for pointing out the two studies on the magnetoresistance with non-magnetic electrodes. We have cited the two papers in the revised manuscript as new references #41 and #42. Both studies used the setup of mechanical break junction, where the local environment of the molecules is not easily characterized at the atomic scale. Here we demonstrate for the first time the tunable magnetoresistance in an atomically controlled environment, where the local environment can be determined in detail by the atomic-scale imaging capability and local spectroscopic characterization of STM/STS, and thus much more information can be obtained. For example, in our current study, we used the STM tip as a mobile electrode, allowing us to study the spatial distribution of the Kondo resonance. This information at the atomic scale is crucial to understand the change of the magnetic states of FePc.

(2) *The novelty of the article rests in the orbital dependent mechanism. Authors provide the change in spatial distribution of the Kondo resonance as the proof of such mechanism and support this interpretation by DFT calculations. From my point of view, these arguments are largely insufficient to justify the main claim of the article, so I suggest rejecting it.*

Reply: We thank the reviewer for recognizing the novelty of an orbit-dependent mechanism of the ensuing magnetoresistance. The change in the spatial distribution of the Kondo resonance, as pointed out in our manuscript, is due to a spin flip transition (or crossover) driven by the applied magnetic field, which turns the magnetic moment of FePc molecules from the direction parallel to the Au(111) surface to the direction perpendicular at roughly 4 T. This flip of the magnetic moment does not change much the system energy, but it does change quite significantly the density distribution of electrons on the two most relevant orbitals, i.e. d_{z2} and dπ, within a few meV energy scale around the Fermi level. This change leads to the change in the spatial distribution of the Kondo resonance, as observed in our measurements. It doesn't result from a direct transition between the two orbitals.

In fact, we initially considered the possibility of the direct transition induced by Zeeman effect. However, after evaluating the PDOS of the ground state, we found that the energy difference between the relevant orbitals was too large to be relevant, as correctly pointed out by the referee. We also found the exchange energy between the molecule and the surface states is too small to be responsible for the effect we observed. We performed noncollinear DFT calculations for a range of magnetic configurations, which took about 500,000 CPU hours, and found the two lowest-energy configurations whose energies are very close and can be switched from one state to another by a small perturbation. As revealed unambiguously by our calculation results, it is the reorientation of the magnetic moment that is responsible for the orbital switching.

(3) *Zeeman effect is associated with tiny energy splitting in comparison with orbital energy differences. Their ground state is not degenerate, so orbital energy differences in the range of meV cannot be present. This contradicts the necessity of having the d_z2 and d_pi orbitals in close proximity to allow crossover.*

Reply: We agree with the reviewer that a Zeeman effect is too small in comparison with the orbital energy differences. But, as mentioned previously this is not relevant to the physical picture described in our manuscript.

Our numerical simulation indicates clearly that the low-energy density distribution of the two most relevant *d* orbitals around the Fermi level depends strongly on the orientation of the magnetic moment. Similar effect has also been observed in Co atoms on an Fe film [PRL 110, 037202 (2013)], where the hybridization between different spin states vanishes when the magnetization is perpendicular to the surface while it exhibits a maximum when the magnetization becomes parallel.

The magnetic anisotropy energy, namely the energy difference between the states with the moment pointing along the in-plane and out-of-plane directions, is about 1 meV for FePc on Au according to our calculations. This milli-eV energy difference for different magnetic moment direction was also obtained independently by other groups. For example, in Phys. Rev. Lett. 110, 097202 (2013), Hu and Wu report a −1.24 meV and ~0.50 meV E_{MAE} for free FePc and FePc on the O site of O-Cu(110) surface. From X-ray magnetic circular dichroism (XMCD) measurements of FePc thin films on an Au substrate with FePc lying flat on the substrate, J. Bartolomé *et al.* estimated the magnetic anisotropy energy to be about 0.53 meV [see equation (7) in Phys. Rev. B 81, 195405 (2010)].

In order to further clarify the effect of the magnetic field on the orbital characteristics of the ground state of the FePc molecule on Au(111), we have added the following sentences to the manuscript:

"*The change of orbital characteristics results from the reorientation of the Fe magnetic moment induced by the applied magnetic field. This reorientation changes the orbital character through spin-orbit interaction*[29]*. The applied magnetic field flips the magnetic moment of Fe from the in-plane to the out-of-plane direction when the Zeeman energy is larger than the magnetic anisotropy energy.*"

"*Similar energy scale of this magnetic anisotropy energy was obtained by other simulations*[30] *as well as by the X-ray magnetic circular dichroism measurements*[31]."

(4) *Even if the state were degenerate, a single reference method (as non-collinear DFT) will not map to the relevant state manifold for this case. In this way, the calculations presented by the authors are not conclusive support for the orbital switching mechanism.*

Reply: As already mentioned, the change of the orbital character does not rely on the degeneracy of d-orbitals. What we calculated is the ground state electronic structures with specific magnetic orientations. It is not a DFT simulation including the calculation of the excitation states, to which the term "single reference method" is referred [see Chem. Rev. 105, 4009 (2005)]. The size of the system we have simulated is very large. Currently it is still beyond computationally feasible to carry out a multi-configurational simulation for this system. The simulations presented in our manuscript are the state-of-art first-principles calculations. It is a well adopted method for evaluating the magnetic anisotropy energy, see for example Phys. Rev. Lett. 110, 097202 (2013).

Response to Reviewer #2

(1) *The authors present a very interesting experimental and theoretical work on the Kondo effect in FePc molecules adsorbed on Au(111). Depending on the adsorption site, a clear Kondo effect visible as a dip at zero bis was found. Varying the temperature, a Kondo effect is confiremd. Interestingly, the Kondo resonance reacts to a magnetic field in a peculiar way. At about 4 T, it vanishes and at higher fields reappears as a peak that splits in accord to a Kondo effect.*

The paper is well written und clearly organized. Where necessary, additional information is given in the supplementary. Overall, the paper is scientifically sound and interesting and should be published in Nature Comm.

Reply: We thank the reviewer for his/her affirmative comments and recommendation for publication.

(2) *Before acceptance, I suggest to go through the paper and improve the English a bit.*

Reply: We have carefully revised the paper to improve the English.

(3) *I suggest to weaken the claim about the shape of the orbitals. While at high fields, I agree that the Kondo cloud looks basically round, the shape at low field is not exactly elliptical (see for example Fig. S3a). I would also suggest to remove the oval and circle in the figures, as they rather suggest a shape and distract the reader from making his/her owen judgement on the shape. The shape of the orbital does change but I would not go so far as to identify the shape in the experiment. The theory shows the orbitals and they kind of agree with the observation. To my taste, this is enough.*

Reply: We followed the reviewer's suggestions and weakened the claim about the shape of the orbitals. In particular, we removed the circles and ovals in the panels of Figs. 3a, 3d, S3b and S3f, and replaced '*elliptical*' by '*non-circular*' in the revised manuscript.

Reviewers' comments:

Reviewer #1 (Remarks to the Author):

In their answer Yang et al. insist in the orbital switching mechanism for the explanation of the observed magnetic field dependence of the Kondo peak. My concerns about the consistency of this explanation are not addressed directly.
Assuming a small anisotropy for the system (as authors do), a more reasonable explanation is magnetic moment reorientation inside the ground ms manifold. This reorientation does not change the orbital composition of the wave function since the ground state is well isolated (and must be since they are assuming a 3A2g ground state. Kondo is a resonant phenomenon, so it is expected to be sensitive to small changes in state alignment.

Such modifications of the Kondo resonance are already reported in literature (see, for instance J. Phys.: Condens. Matter 27 (2015) 113202).

Some of the references about similar systems do not apply in this case, for instance, the ground state analyzed in Bartolome's PRL paper is different from the one assumed here and the paper about Co atoms in gold is characterized by large unquenched angular momentum in the ground state. Conclusions from these papers are cannot transferred to this case.

In sum, I do not think there is enough evidence to sustain the proposed mechanism. Furthermore, there is a more likely explanation which is not explored by the authors.

Reviewer #2 (Remarks to the Author):

The authors have improved the manuscript and have, to my opinion, answered to the questions of both referee convincingly.
I support publication of this work.

List of Changes (marked by the red color in the revised text)

Main text:

1. We have added several sentences to emphasize the role of orbital symmetries in determining the spatial distribution of the Kondo resonance:

"*The change of spatial distributions of Kondo resonance can be understood by considering the different spatial symmetries of the* d_π *and* d_{z^2} *orbitals (Fig. 1). The hybridization of* d_π *orbital with the neighbouring atoms inside the FePc molecule results in a more extended Kondo resonance distribution at low field; while the* d_{z^2} *orbital, due to its different symmetry, hybridizes less with the molecular orbitals, giving rise to the more localized spatial distribution of Kondo resonance at high magnetic field.*"

2. We have added one sentence to clarify that Zeeman energy doesn't induce direct orbital transitions:

"*Note that the reorientation of the magnetic moment doesn't mean that the Zeeman energy induces direct orbital transitions, since only the low-energy density distribution of the d orbitals near* E_F *changes.*"

3. We have added several sentences to examine another possible explanation of the change of Kondo resonance:

"*A level crossing within the low-energy spin multiplet with quenched orbital angular momentum cannot explain the change of the spatial distribution of the Kondo resonance with increasing magnetic field, since this scenario would only result in a tiny change of the orbital composition in the wave function.*"

4. Old reference 31: [J. Bartolomé et al. Phys. Rev. B 81, 195405 (2010)] is removed, and correspondingly "... *as well as by the X-ray magnetic circular dichroism measurements*[31]." is removed.

5. Format changes to comply with format requirements of Nature Communications.

(a) Ref. [1] in the old abstract is moved to in the 1st sentence of introduction.

(b) The 3rd sentence of the old abstract

"The molecular spin states can be controlled mechanically[3]*, electrically*[4]*, or by magnetic field*[4,9,10]*, providing a useful handle to tune the flow of current. For example, using a magnetic field, novel spin-dependent transport phenomena can be tuned at the single-molecule level such as the negative differential resistance*[9] *or the highly-correlated Kondo effect*[10]*."*

is moved to the introduction as the 2nd sentence.

(c) Several changes in wording to reduce the length of Abstract to less than 150 words.

(d) We added subheadings in the Results section.

(e) The old 1st section in supplemental information is moved to the main text as Methods section.

(f) Conflict of interest statement is added.

(g) Other minor changes to comply with format requirements of Nature Communications.

Response to Reviewer #1

Comment: *My concerns about the consistency of this explanation are not addressed directly. … Assuming a small anisotropy for the system (as authors do), a more reasonable explanation is magnetic moment reorientation inside the ground ms manifold. This reorientation does not change the orbital composition of the wave function since the ground state is well isolated (and must be since they are assuming a 3A2g ground state. Kondo is a resonant phenomenon, so it is expected to be sensitive to small changes in state alignment.*

Reply: We thank the reviewer for the careful reading of the manuscript and the comment. However, we do not agree with this comment, and feel that the reviewer might have overlooked or misunderstood the arguments we gave in the manuscript as well as in our previous reply. In fact, our mechanism was found based on a thorough analysis of experimental observation combined with density functional first principles calculations. We believe our analysis is comprehensive and convincing.

(1) The pure "*magnetic moment reorientation*" without orbital redistribution was also the first mechanism we tried to understand the experimental results. However, this picture cannot explain the dramatical change in the spatial distributions of the Kondo resonances from low to high field --- the spatial distribution becomes more localized around the Fe ion at higher magnetic fields. This phenomenon, nevertheless, can be readily understood by considering the different spatial symmetries of the two most relevant *d* orbitals: the hybridization of d_π orbital with the neighbouring atoms inside the FePc molecule results in a more extended Kondo resonance distribution at low field; while the $d_z{}^2$ orbital, due to its different symmetry, hybridizes less with the molecular orbitals, giving rise to the more localized spatial distribution at high magnetic field.

(2) The Zeeman energy generated by the applied magnetic field is too small to swap the energy levels of d_π and $d_z{}^2$ orbitals. However, the change of the orientation of the magnetic moment may change significantly the relative weights of these two

orbitals on the Fermi surface due to the spin-orbit coupling. This is because the spatial orientations of the density distribution of these two orbitals, hence their couplings with the magnetic moment (especially its orientation), are different. It is the change of the low-energy density of states around the Fermi level of these two orbitals by varying the moment orientation that leads to the phenomenon we observed. This picture is confirmed by our DFT calculations which show clearly that the low-energy density distribution of the two most relevant *d* orbitals around the Fermi level depends strongly on the orientation of the magnetic moment.

(3) Our orbital dependent mechanism is also supported by the different line shapes of the Kondo resonance at different magnetic fields. The dip-to-peak transition of the Kondo resonance with magnetic field can be explained by considering the different spatial symmetries of the *d* orbitals, which couple differently with the STM tip.

We added the following sentences in the revised manuscript to emphasize the role of the orbital symmetries in determining the spatial distribution of the Kondo resonance:

"*The change of spatial distributions of Kondo resonance can be understood by considering the different spatial symmetries of the* d_π *and* d_{z^2} *orbitals (Fig. 1). The hybridization of* d_π *orbital with the neighbouring atoms inside the FePc molecule results in a more extended Kondo resonance distribution at low field; while the* d_{z^2} *orbital, due to its different symmetry, hybridizes less with the molecular orbitals, giving rise to the more localized spatial distribution of Kondo resonance at high magnetic field.*"

Comment: *Such modifications of the Kondo resonance are already reported in literature (see, for instance J. Phys.: Condens. Matter 27 (2015) 113202).*

Reply: We thank the reviewer for pointing out this paper. However, the paper mentioned by the referee is a review paper on the magnetic anisotropy in single magnetic molecules. This paper reported the splitting of the Kondo resonance under

magnetic field, which is different from the dip-to-peak transition observed in our current work.

Comment: *Some of the references about similar systems do not apply in this case, for instance, the ground state analyzed in Bartolome's PRL paper is different from the one assumed here and the paper about Co atoms in gold is characterized by large unquenched angular momentum in the ground state. Conclusions from these papers are cannot transferred to this case.*

Reply: We thank the reviewer for this comment. We agree that since the ground state of FePc in [Phys. Rev. B 81, 195405 (2010)] is different from that observed in our system the magnetic anisotropy value is not directly comparable. We have removed Ref. [31] in the revised manuscript.

Response to Reviewer #2

Comment: *The authors have improved the manuscript and have, to my opinion, answered to the questions of both referee convincingly.*

I support publication of this work.

Reply: We thank the reviewer for recommendation for publication.

REVIEWERS' COMMENTS:

Reviewer #1 (Remarks to the Author):

Although the experimental work looks properly done and the topic is indeed interesting, I have fundamental concerns about the physical consistency of their theoretical interpretation. As there is no sensible change in this revision, I am afraid I cannot recommend this paper

Reviewer #4 (Remarks to the Author):

This manuscript reports the observation of magnetic field dependent transport through a single iron phthalocyanine molecule on an Au(111) surface by STM. Kondo resonance line shape and spatial extent of the Kondo resonance are measured as a function of field and temperature. The observed dependencies and the resulting tunable magnetoresistance of up to 93% at 11 T and 0.4 K are explained by the authors to be due to the field-induced reorientation of the magnetic moment of the Fe center. The Fe moment rotates from in-plane at 0 T to out-of-plane at 11 T and thereby alters the electron distribution among the Fe d-orbitals near the Fermi level via spin-orbit interaction from d_π for in-plane moment at 0 K to d_z^2 for out-of-plane moment at 11 T.

The manuscript is clearly structured and well written. The interpretation of the data and the derived conclusions are corroborated by DFT calculation and additional data given in the supplementary material. Minor deficiencies and points of criticism raised by the Reviewers were all corrected and/or taken into account by the authors in the revised manuscript. In particular, the doubts Reviewer #1 about underlying mechanism (change of orbital characteristics) are clearly and convincingly refuted by the authors in their Replies. Corresponding changes to the manuscript clarify the Authors' arguments in favor of their interpretation and also mention and discard the Reviewer's alternative explanation (level crossing within the low-energy spin multiplet). The strongest and convincing arguments in favor the Authors' model are (i) the field-dependent spatial extent of the Kondo resonance, (ii) the straight-forward explanation of the field-dependent evolution of the spectral shape of the Kondo resonances, and (iii) the DFT calculations.

To my opinion, the criticism of Reviewer #1 is clearly refuted, and this scientifically sound and interesting manuscript should be published in Nature Communications.

Response to Reviewer #1

Although the experimental work looks properly done and the topic is indeed interesting, I have fundamental concerns about the physical consistency of their theoretical interpretation. As there is no sensible change in this revision, I am afraid I cannot recommend this paper

Reply: Our theoretical interpretation is based on a thorough analysis of experimental observation combined with density functional first principles calculations. We believe our analysis is comprehensive and convincing.

Response to Reviewer #4

This manuscript reports the observation of magnetic field dependent transport through a single iron phthalocyanine molecule on an Au(111) surface by STM. Kondo resonance line shape and spatial extent of the Kondo resonance are measured as a function of field and temperature. The observed dependencies and the resulting tunable magnetoresistance of up to 93% at 11 T and 0.4 K are explained by the authors to be due to the field-induced reorientation of the magnetic moment of the Fe center. The Fe moment rotates from in-plane at 0 T to out-of-plane at 11 T and thereby alters the electron distribution among the Fe d-orbitals near the Fermi level via spin-orbit interaction from d_π for in-plane moment at 0 K to d_z^2 for out-of-plane moment at 11 T.

The manuscript is clearly structured and well written. The interpretation of the data and the derived conclusions are corroborated by DFT calculation and additional data given in the supplementary material. Minor deficiencies and points of criticism raised by the Reviewers were all corrected and/or taken into account by the authors in the revised manuscript. In particular, the doubts Reviewer #1 about underlying mechanism (change of orbital characteristics) are clearly and convincingly refuted by the authors in their Replies. Corresponding changes to the manuscript clarify the

Authors' arguments in favor of their interpretation and also mention and discard the Reviewer's alternative explanation (level crossing within the low-energy spin multiplet). The strongest and convincing arguments in favor the Authors' model are (i) the field-dependent spatial extent of the Kondo resonance, (ii) the straight-forward explanation of the field-dependent evolution of the spectral shape of the Kondo resonances, and (iii) the DFT calculations.

To my opinion, the criticism of Reviewer #1 is clearly refuted, and this scientifically sound and interesting manuscript should be published in Nature Communications.

Reply: We thank the reviewer for recommendation for publication.
